# European Laryngological Society position paper on laryngeal dysplasia Part I: aetiology and pathological classification

**DOI:** 10.1007/s00405-020-06403-y

**Published:** 2020-10-13

**Authors:** Edward Odell, Hans Edmund Eckel, Ricard Simo, Miquel Quer, Vinidh Paleri, Jens Peter Klussmann, Marc Remacle, Elisabeth Sjögren, Cesare Piazza

**Affiliations:** 1 Head and Neck Pathology, King’s College London, Guy’s Hospital, London, SE1 9RT UK; 2 Department of Oto-Rhino-Laryngology, Klagenfurt General Hospital, Feschnigstr. 11, Klagenfurt, Austria; 3 Department of Otorhinolaryngology Head and Neck Surgery, Guy’s and St Thomas’ Hospital NHS Foundation Trust, London, UK; 4 Department of Otorhinolaryngology and Head and Neck Surgery, Hospital de la Santa Creu i Sant Pau, Universitat Autònoma de Barcelona, Barcelona, Spain; 5 Head and Neck Unit, Royal Marsden Hospital, London, UK; 6 Department of Otorhinolaryngology, Head and Neck Surgery, Medical Faculty, Cologne, Germany; 7 Department of Otorhinolaryngology, Head and Neck Surgery, CH Luxembourg, Luxembourg, Belgium; 8 Department of Otorhinolaryngology, Head and Neck Surgery, Leiden University Medical Center, Leiden, The Netherlands; 9 Department of Otorhinolaryngology- Head and Neck Surgery, ASST Spedali Civili of Brescia, University of Brescia, Brescia, Italy

**Keywords:** Carcinoma in situ, Dysplasia, Laryngeal intraepithelial neoplasia, Laryngeal carcinoma

## Abstract

**Purpose of review:**

To give an overview of the current knowledge regarding the aetiology, epidemiology, and classification of laryngeal dysplasia (LD) and to highlight the contributions of recent literature. As most cases of dysplasia occur at the glottic level and data on diagnosis and management are almost exclusively from this location, laryngeal dysplasia in this position paper is taken to be synonymous with dysplasia of the vocal folds.

**Summary:**

LD has long been recognized as a precursor lesion to laryngeal squamous cell carcinoma (SCC). Tobacco and alcohol consumption are the two single most important etiological factors for the development of LD. There is currently insufficient evidence to support a role of reflux. Although varying levels of human papillomavirus have been identified in LD, its causal role is still uncertain, and there are data suggesting that it may be limited. Dysplasia has a varying presentation including leukoplakia, erythroleukoplakia, mucosal reddening or thickening with exophytic, “tumor-like” alterations. About 50% of leukoplakic lesions will contain some form of dysplasia. It has become clear that the traditionally accepted molecular pathways to cancer, involving accumulated mutations in a specific order, do not apply to LD. Although the molecular nature of the progression of LD to SCC is still unclear, it can be concluded that the risk of malignant transformation does rise with increasing grade of dysplasia, but not predictably so. Consequently, grading systems are inherently troubled by the weak correlation between the degree of the dysplasia and the risk of malignant transformation. The best data on LD grading and outcomes come from the Ljubljana group, forming the basis for the World Health Organization classification published in 2017.

## Aetiology

Carcinogenesis is traditionally considered to be a multiyear, multistep, multipath disease of progressive genetic damage but this view is challenged for cancers arising in dysplasia [1, 2]. Aetiological factors involved in the genesis and progression of laryngeal dysplasia (LD) and squamous cell carcinoma (SCC) include environmental factors (carcinogen exposure), genetic changes, epigenetic aberrations and immune escape. Tobacco and alcohol consumption are the most important single factors, implicated in 75% of all head and neck SCC (HNSSC) and have a multiplicative combined effect [2]. Additionally, the role of occupational factors have also been assessed [3]. From a recent review of the literature, the authors conclude that there is insufficient evidence to support a causal role of reflux in laryngeal SCC, mainly because of the confounding effect of tobacco and alcohol consumption and the inaccuracies in the diagnosis of reflux [4]. Human papillomavirus (HPV) infection is now considered a potential aetiological agent and will be covered separately in a following section. Precancerous laryngeal lesions occur with an estimated annual incidence in the United States of 10.2 lesions per 100,000 males and 2.1 lesions per 100,000 females [5]. The current annual incidence of LD in Europe is not known.

## The role of HPV in LD

More than 200 different HPV genotypes have been distinguished and those can be divided into low-, intermediate-, and high-risk types. Recurrent laryngeal papillomatosis is primarily caused by low-risk HPV types 6 and 11. The risk of malignant transformation in laryngeal papillomatosis is reported to be 1–7% [6]. In the paediatric population earlier onset of papillomatosis and pulmonary disease are predictors of dysplastic transformation whereas in adults the risk is higher for patients with later onset [7]. In a large German series, the incidence of airway carcinoma in recurrent respiratory papillomatosis (RRP) was 4% [8]. The rate for dysplasia (mild to moderate) was 7.5% for the juvenile-onset RRP, 12.1% for adult-onset RRP and 19.7% for single papillomas. Most dysplasias were located at the glottic region [8]. HPV-DNA integration in RRP can be related to malignant transformation [9]. Rates of dysplasia in laryngeal papillomatosis vary from 5 to 28% [7, 8, 10], whereas prevalence of HPV in low- and high-grade dysplasia varies between 0 to 83% [10, 11]. HPV types that have been detected are 6, 11, 16, 18, 33, and 57. Davids et al. investigated 24 patients with laryngeal papillomatosis and LD and found HPV 6/11 in 50% of patients with low-grade dysplasia and in 88% of patients with high-grade dysplasia [10]. No HPV high-risk types were detected by these authors when screened for HPV 16 and 18 [10]. Waters et al. investigated precancerous lesions of the larynx for HPV high-risk types and only 1 out of 15 resulted positive by in situ hybridization technique [12].

Pagliuca et al. analyzed 30 samples with histologically proven LD from smokers and non-smokers by polymerase chain reaction (PCR). HPV-DNA was not detected in any (0%) of the samples [11]. Though the association between LD and HPV is well-known, the correct prevalence should be confirmed in larger, prospective series of patients evaluated by multiple DNA and RNA testing, because DNA-PCR alone might overestimates the proportion of virus driven LD [13].

In other entities such as cervical carcinoma, HPV-associated premalignant lesions are well documented, whereas in oropharyngeal SCC this phenomenon is lacking [14]. Only a few studies with small numbers of patients have investigated the role of HPV in premalignant lesions of the larynx and detection of high-risk types was lacking. There is a fundamental difference between HPV-DNA detection in the healthy population, LD and laryngeal cancer, and the rates of biological activity of HPV-DNA often remains unclear. Higher rates of HPV detection seem more likely to be related to transient HPV infections and future studies are necessary to clarify the impact of HPV in pathogenesis.

## Clinical presentation

LD usually occur at the level of the vocal cords. In the supraglottis and/or subglottis it is usually asymptomatic and diagnosed only incidentally during examinations performed for other reasons. The guiding symptom of LD, like for any other pathology at the level of the vocal cords, is an altered voice production. Since this symptom is unspecific and can be observed in all pathological alterations of the vocal cords, it simply indicates the need for a proper laryngeal evaluation, to be performed by an otolaryngologist using at least a transnasal (or transoral) fiberoptic instrument. Pain, swallowing disorders or airway problems are rarely encountered in LD. At laryngoscopy, leukoplakia, erythroleukoplakia, hyperkeratotic lesions, mucosal reddening or thickening with exophytic tumour-like alterations may all be found in LD and no single clinical diagnosis should be considered pathognomonic for LD. However, that being said, LD frequently presents as laryngeal leukoplakia or hyperkeratosis. In a comprehensive review of the literature, Isenberg et al. found no dysplasia in 53.6% out of 2188 laryngeal leukoplakia biopsies [15]. Mild and/or moderate dysplasia and carcinoma in situ were present in 33.5% and 15.2% of biopsies, respectively. SCC developed in 3.7%, 10.1%, and 18.1% of patients whose initial biopsies demonstrated no dysplasia, mild to moderate dysplasia, or severe dysplasia and carcinoma in situ.

## Classification

Clinicians often do not understand why dysplasia grading cannot be easily standardised. The reasons lie in the very varied histological appearances, confounding non-specific changes such as inflammation, lack of understanding of the underlying process and the difficulty of proving validity of grading schemes through long term outcome studies. Methods for grading LD were initially influenced heavily by those used in the cervix, where dysplasia progresses from the basal layer to the surface. However, this is inappropriate in all upper aerodigestive tract mucosa where the mucosa keratinises and changes are driven by tobacco carcinogens and not HPV integration in the basal layers. Application of cervical dysplasia scoring systems significantly under-grades laryngeal dysplasia. However, these old concepts remain in both oral and laryngeal dysplasia grading systems.

There is no agreement on the nature of the spectrum of changes seen in precursor lesions, some considering all as dysplastic, while others accepting hyperplasia as an initial stage in carcinogenesis. A particular problem is the definition of carcinoma in situ, and whether it can be identified and merits a separate designation.

The reference standard for any dysplasia grading system must be the clinical outcome. Unfortunately outcome studies are difficult to perform and require long follow up periods. For this reason many published papers concentrate either on case control series or on interobserver agreement. Case control studies have merit but suffer the problem that the tissue being examined microscopically is not the area that became malignant. Outcome studies can improve on this by including multiple biopsy samples over many years.

Published papers on interobserver agreement often suggest that dysplasia grading is inaccurate and of limited clinical value of limited clinical value. This negative impression arises largely through analysis using the kappa statistic, which is inappropriate for grading continuum [16]. Using this analysis for dysplasia grading, it is almost impossible to prove better than ‘moderate’ agreement between pathologists.

The histological changes of dysplasia form a continuous spectrum and it is not expected that pathologists will be able to agree on an exact grade of dysplasia in every case. Despite this, data from interobserver agreement studies are used to promote binary rather than 3 or 5 grade scales. It is evident that if there are more grades of dysplasia, then there are more boundaries between grades over which the pathologists can disagree. Conversely, a grading system that only had one category would be completely reproducible, but useless clinically. It is also important to remember that it is possible to be reproducibly wrong. Disagreement between pathologists provides more information about the correct degree of dysplasia than agreement [17], so that for published studies in oral dysplasia having multiple observers is considered the gold standard method. Integration of different observations is usually done by consensus, involving a third observer if required, as described by Speight et al. [18].

The best data on LD grading and outcome comes from the Ljubljana group who have published over many years, now with over 30 years follow-up data [19]. Unfortunately, during that period their grading system has changed its terminology and its difficult definitions have led to it being adopted in only a few centres. Its excellent evidence base has led to this system forming the framework of the World Health Organization (WHO) system published in 2017 [20]. This divides LD into only two categories of low-grade and high grade dysplasia. The WHO book indicates that a category of carcinoma in situ may still be used but the criteria, ‘complete loss of stratification and/or severe cytological atypia and atypical mitoses’ allow considerable flexibility in their application. This additional category allows pathologists to provide a diagnosis of malignancy rather than dysplasia and may be helpful in centres where radiotherapy (RT) is used for these most severe changes. Table 1 shows the evolution of the WHO grading system for LD.

**Table 1:** Comparison of grading systems for LD, after WHO 2017 [20], with corrected levels. The published version limits low-grade dysplasia to the lower third, rather than the lower half of the epithelium

Level of abnormal maturation (WHO 2005)	WHO 2005 Classification [34]	SIN Classification [35]	Ljubljana Classification [33]	Amended Ljubljana classification [19]	WHO 2017 [20]
	Squamous hyperplasia	Squamous hyperplasia	Squamous hyperplasia	Low grade SIL	Low grade dysplasia
Lower 1/3	Mild dysplasia	SIN 1	Basal/parabasal hyperplasia		
1/3 to 1/2	Moderate dysplasia	SIN 1 or 2	Atypical hyperplasia	High grade SIL	
Upper 1/2–3/4		SIN 2			High grade dysplasia *
Full thickness	Severe dysplasia				
	Carcinoma in situ		Carcinoma in situ	Carcinoma in situ	

*SIN* squamous intraepithelial neoplasia, *SIL* intraepithelial lesion

^*^A grade of carcinoma in situ may be used if a three-tiered system is preferred

Recent grading systems places emphasis on architectural changes but still define the difference between dysplasia grades primarily in terms of the thickness of the epithelium involved, up to half the thickness being considered low-grade provided the upper layers have normal maturation. The effect of this definition is to ensure that hyperplasia, inflammatory and reactive changes are safely grouped into the low-grade lesions. However, it leaves most truly dysplastic lesions in a single group without identifying the highest risk ones and cannot clearly differentiate low-risk from non-risk lesions [21]. If follow-up studies using a 3/4 grade system published since 2000 are compared, it is striking that within any individual study there is not a good correlation between the risk of malignant transformation and the degree of dysplasia, but that the mean values show good correlation in metanalysis [22]. It can be concluded that the risk of malignant transformation does rise with increasing grade of dysplasia, but unpredictably so.

From the clinical point of view, it is natural to look to the published data to identify risk of malignant transformation. However, transformation will only occur in a minority of even high-risk lesions and may take many years to develop.

## Lessons learned from oral dysplasia

It has not proved possible to define a single epithelial dysplasia grading system applicable to all HN sites [20], because the nature of the diseases causing dysplasia at different sites vary, as do the histological changes. For the oral cavity, the WHO system continues to recommend a three grade division into mild, moderate, and severe dysplasia and considers a two grade system unproven [20]. Carcinoma in situ is considered synonymous with severe dysplasia.

Dysplasia in larynx differs from other upper aerodigestive tract sites such as mouth for many reasons including exposure to tobacco carcinogens in only the vapour phase, and induction of four times as many mutations per cell in larynx than oral cavity [23]. In addition there are a number of oral potentially malignant disorders that are not necessarily associated with smoking. Oral lesions also show a greater range of cytological atypia in low-grade dysplasia than laryngeal lesions [24] and severe dysplasia can affect only the lower third of the epithelium.

Reviewing the outcome data for the Ljubljana/WHO 2017 system shows the overall risk for even a high-grade lesion in the larynx is relatively low when compared this with the equivalent situation in the oral cavity. Oral severe dysplasia has more than three times the risk of malignant transformation at 15 years as compared to laryngeal high grade dysplasia [19, 20, 25, 26].

The molecular nature of oral lesions is much better understood than laryngeal lesions. For the oral cavity, the traditional hypothetical pathways to cancer involving accumulated mutations [27] in a specific order are wrong. We now understand that the genetically damaged cells in dysplastic oral epithelium accumulate genetic changes randomly and do not form a single clone that spreads laterally to colonise the tissue. Rather, numerous clones of cells coexist within a dysplastic epithelium, each with different types of mutation, gene inactivation profile, and chromosomal damage. Clones are probably interdependent both on each other and on the normal cells for some growth factors, nutrients, and survival signalling. This evolutionary model of dysplasia is established in Barrett’s oesophagus [28] and the mouth [29–31] and has a number of important consequences for our understanding of the development of cancer.

Dysplasia, at least in the mouth, is not a process that is progressing to cancer but, rather, the result of random mutations and chromosomal changes that generate primed cells susceptible to further genetic changes. In a seminal study, Makarev et al. performed pathway analysis to identify which of the many cancer-related signalling pathways were involved in oral cancers and precursor lesions [32]. They showed that cancer was associated with activating mutations in one of five main pathways, the AKT/mTOR, RAS/RAF/MAPK, JAK/STAT, WNT or TGF beta pathways. When individual pathways were examined in cancers and precursor lesions, it was striking that the latter showed inhibition of almost all pathways studied, while the cancers showed pathway activation. The only pathways showing activation in dysplastic lesions were those associated with apoptosis and cell death.

Interestingly, 20 of 68 precursor lesions had carcinoma-like genetic changes but, even in this minority, only 7 progressed to cancer in a median period of six years. Twenty-eight other precursors progressed to cancer without developing cancer-like molecular changes first. These workers and others working on exome sequencing have all concluded that there is no predictable genetic pathway to carcinoma in the oral cavity. Clinicians need to develop an understanding that dysplastic changes are not the start of a relentless progression to cancer.

## Conclusions

LD has long been recognized as a precursor lesion to laryngeal SCC. Tobacco and alcohol consumption are still to be considered the single most important etiological factors for the development of LD, while the role of acidic reflux and HPV need to be investigated in deeper details. Dysplasia may have varying clinical presentations including leukoplakia, erythroleukoplakia, mucosal reddening or thickening with exophytic, “tumor-like” alterations, none of which is pathognomonic of a given histopathologic nature. About half of leukoclastic lesions will contain some form of dysplasia. However, it has become clear that the traditional pathways to cancer, involving mutations progressively accumulated in a specific order, do not hold any more for LD. Although the molecular nature of the progression of LD to SCC is still unclear, it can be safely concluded that the risk of malignant transformation rises with increasing grade of dysplasia, but not predictably so. Consequently, the adopted grading systems continue to be inherently troubled by the weak correlation between the degree of the dysplasia and the risk of malignant transformation.
